# Are dental visiting patterns and oral pain associated with dental disease among Norwegian adults? A cross‐sectional study based on the Tromsø study

**DOI:** 10.1002/cre2.753

**Published:** 2023-07-06

**Authors:** Hege Nermo, Elin Hadler‐Olsen

**Affiliations:** ^1^ The Public Dental Health Competence Center of Northern Norway Tromsø Norway; ^2^ Department of Clinical Dentistry, Faculty of Health Sciences UiT The Arctic University of Norway Tromsø Norway; ^3^ Department of Medical Biology, Faculty of Health Sciences UiT The Arctic University of Norway Tromsø Norway

**Keywords:** caries, dental care, pain, periodontitis

## Abstract

**Objectives:**

The present study aims to describe the dental visiting patterns in a Norwegian adult population and their associations with sociodemographic and oral health variables, including oral pain. We further explore if the utilization of dental health services and oral pain predicts caries and periodontitis, the most common oral diseases.

**Materials and Methods:**

We use data from the seventh wave of the Tromsø study performed in 2015–2016. In this cross‐sectional survey, all residents 40 years or older in Tromsø municipality in Norway were invited, of whom 21,083 (65%) participated. All participants answered questionnaires assessing sociodemographic characteristics, use of health services, and self‐reported health measures, including pain. Almost 4000 participants underwent a dental examination with registration of caries and periodontitis. Associations of dental visiting patterns and utilization of dental services the past 12 months with sociodemographic‐, self‐reported‐, and clinical oral health measures were analyzed by cross‐tabulation and Pearson's *χ* ^2^ tests, as well as with logistic regression analyses with caries and periodontitis as outcomes.

**Results:**

A regular, annual dental visiting pattern was the most common, but among respondents with severe dental anxiety and poor dental health, visiting for acute problems only or never (symptomatic visiting) was the most common. Intervals of more than 24 months between visits and a symptomatic visiting pattern were associated with caries, whereas shorter than 12‐month intervals and a symptomatic visiting pattern were associated with periodontitis. Many characteristics were shared among respondents with the lowest and the highest utilization of dental services, including oral pain, a difficult financial situation and poorer self‐reported and clinical dental health.

**Conclusions:**

Regular dental visits at 12–24 month intervals were associated with beneficial oral health parameters, compared with more frequent, rarer, and symptomatic dental visiting patterns. Oral pain was an unreliable predictor of caries and periodontitis.

## 1 BACKGROUND

In Norway, most health services are utilized in response to a perceived need, whereas current dental health service guidelines recommend regular screening and prevention visits. The high prevalence of oral diseases has justified the focus on screening in dental health services (Kassebaum et al., 2017). Oral diseases are the most widespread of all conditions globally (2022) and they were the ninth most important cause of health loss in Norway in 2016, measured in years lived with disability (Øverland et al., 2018). The leading causes of health loss due to oral diseases are caries in permanent teeth, severe periodontitis, and tooth loss (Kassebaum et al., 2017), where tooth loss is often a sequela of the two former diseases. As both caries and periodontitis may be asymptomatic until the damage to the tooth or the supportive tissue is severe (Selwitz et al., 2007), regular screening is indicated. In Norway, dental health services are free until the age of 19 years and partly sponsored for young adults between 19 and 24 years, after which they are paid out‐of‐pocket with few exceptions. Whereas children and adolescents mostly have their services in public dental clinics, most adults receive dental health services from private practitioners.

Ideally, dental visiting frequencies should be individualized and reflect the current disease risk (Selwitz et al., 2007). However, the dental visiting pattern may be affected by more than a professional risk assessment. The utilization of dental services varies across factors such as the availability of the services, personal finances, and dental anxiety (Hadler‐Olsen & Jönsson, 2021; Holde et al., 2018; Jonsson et al., 2020). The perceived need for treatment may rely on pain and functional limitations, with less awareness of the need for secondary preventive measures, changes in risk factors over time, and the presence of early stages of diseases. Several clinical oral health measures have improved in the population over the past 50 years (Frencken et al., 2017; Norderyd et al., 2015). Although this is good news for most, studies have suggested that dental practitioners in private practice already have and will continue to experience, fewer regular patients, and a loss of income (Grytten & Skau, 2021). More troubling are findings, indicating that dentists set their patients to more frequent recall intervals based on economic indices over clinical risk assessments (Grytten et al., 2022). The inference that private practitioners have a questionable professional justification for recall intervals leaves important questions unanswered concerning the need for regular dental check‐ups and whether the frequency of dental visits reflects the prevalence of dental disease.

The present study aims to describe the dental visiting pattern in a Norwegian adult population and its association with sociodemographic, self‐reported, and clinical oral health variables, with a special focus on the characteristics of those with high demands for dental services. As regular dental visits aim to promote oral health, we finally analyze the associations of dental visiting patterns and oral pain with dentin caries and marginal periodontitis while controlling for sociodemographic variables.

## 2 METHODS

### 2.1 Study design and participants

In this cross‐sectional study, we used data from the seventh survey of the Tromsø Study (Tromsø 7) carried out in 2015–2016. All adults 40 years or older in Tromsø municipality (*n* = 32,591) were invited by mail, of whom 21,083 (65%) participated. All participants answered questionnaires assessing sociodemographic, general‐, mental‐, and oral health‐related variables. A random selection of the participants (*n* = 3942) were offered a dental examination and all but three accepted (*n* = 3939). The dental examination included full‐mouth periodontal probing depth and registration of bleeding on probing at four sites per tooth, four bitewing, and one panoramic radiograph, as well as eight intraoral digital photos. The dental examination was performed by six dental hygienists whom an experienced periodontist trained before the study. Periodontal measurements were calibrated twice, as previously described (Petrenya et al., 2022). Calibration was done on four to six teeth at four to six sites per tooth and the median intraclass correlation coefficient was 0.79 for the first round of calibration and 0.82 for the second round. Bone level on panoramic radiographs was measured by three different examiners. The examiners were calibrated for the specific measurements twice, and the median intraclass correlation coefficient was 0.76 on both occasions. Caries was diagnosed based on bitewing radiographs and clinical photos by seven experienced and calibrated dentists. The calibration and caries registration protocol has been described in detail previously (Mulic et al., 2020). In brief, they assessed primary occlusal and proximal caries and secondary caries on a 5‐graded scale, D1–5, where D1–2 signifies caries involving enamel, and D3–5 signifies caries involving dentin (Amarante et al., 1998). For the current study, we have defined caries as D3–5. The mean interexaminer agreement for the caries registration was 0.70 and 0.81 for registrations based on radiographs and clinical photos, respectively, as determined by weighted Cohen's *κ*. The Tromsø study was approved by the Norwegian Data Protection Authority (14/01463‐8/CGN), and the Regional Committee for Medical and Health Research Ethics approved the present study (320778).

### 2.2 Variables

#### 2.2.1 Sociodemographic variables

Sex and age were obtained from the National Population Register. Age was categorized into the following: (1) 40–54 years; (2) 55–69 years; and (3) 70 years or older in the analyses. The highest level of completed education was assessed by one question with four options: primary school 10 years or less; secondary school 3 years or more; higher education <4 years; higher education 4 years or more. For analyses, we dichotomized the variable into (1) no higher education and (2) higher education. The respondents rated their current financial situation on a five‐point scale; very good, good, average, difficult, or very difficult, which we recoded into (1) good (very good or good); (2) average; and (3) difficult (difficult or very difficult).

#### 2.2.2 Self‐reported oral health variables

Respondents were asked if they had regular dental care (dental visiting pattern) with the options: (1) I regularly visit more than once a year; (2) I regularly visit once a year; (3) I regularly visit every second year; (3) I regularly visit with longer intervals than every second year; (4) I visit for acute problems only; and (5) I never visit. For analyses, options 4 and 5 were merged into one category that was named symptomatic visiting. Respondents were also asked about how many times they had visited a dentist or a dental hygienist in the preceding 12 months. For analyses, we categorized the answers into (1) no visits; (2) one visit; (3) two visits; (4) three or four visits; and (5) five or more visits. Oral pain was assessed by the Graphical Index of Pain (Steingrimsdottir et al., 2020). This is an online tool where the respondents are presented with a body map with 10 first‐tier regions, one of them being the head. They mark each region where they have experienced pain during the last 4 weeks, excluding transient, brief pain, and menstrual pain. Marked first‐tier regions are magnified into anatomical sites at a second tier where the respondents mark all second‐tier sites at which they have experienced pain. In the current study, we have included pain in the following second‐tier regions of the head: the temporomandibular joints, the teeth, the gums, the oral mucosa (including tongue and throat), and the lips, because pain in these regions are commonly handled in the dental practice. Pain was coded as 0 (no pain) or 1 (pain) for each of the sites included. We summarized pain in these regions (termed oral pain) and dichotomized it into: (1) no pain and (2) pain in at least one region. Respondents rated their dental health on a five‐grade scale from very poor to very good. For analyses, we categorized the variable into (1) poor dental health; (2) moderate dental health; and (3) good dental health. Dental anxiety was assessed by the validated, Norwegian version of the Modified Dental Anxiety Scale (Haugejorden & Klock, 2000; Humphris et al., 2009), which comprises five questions to assess dental anxiety, rated on an ordinal scale from 1 to 5. The sum score for all questions (range: 5–25) were dichotomized, where a score of ≥19 was defined as high dental anxiety (Humphris et al., 2009).

#### 2.2.3 Clinical oral health variables

Dentin caries was assessed as described above and categorized into: (1) no caries; (2) one carious tooth; (3) two carious teeth; (4) three or more carious teeth. Periodontitis was assessed as described above and diagnosed according to the Centres for Disease Control/American Academy of Periodontology (Eke et al., 2012; Page & Eke, 2007), categorized into (1) no periodontitis; (2) mild periodontitis; (3) moderate periodontitis; and (4) severe periodontitis. In regression analyses, caries was dichotomized into (0) no dentine caries (1) at least one dentine caries, and periodontal status was dichotomized into (0) no or mild periodontitis and (1) moderate or advanced periodontitis. The rational for this dichotomization is that having dentin caries or moderate or advanced periodontitis suggest a need for treatment or closer follow‐up.

### 2.3 Statistical analyses

We used the Statistical Package for Social Sciences for Windows version 26 (IMB Corporation) for statistical analyses. Dental visiting pattern and number of dental visits in the past 12 months were cross‐tabulated against the sociodemographic, self‐reported oral health, and clinical oral health variables. The statistical significance of differences between groups were assessed by Pearson's *χ* ^2^ tests. Logistic regression was used to assess the association of oral pain and dental visiting patterns with caries and periodontitis when controlling for sex, age, personal finances, tooth loss, and dental anxiety.

## 3 RESULTS

### 3.1 Dental visiting pattern

A regular, annual visiting pattern was the most common (Table 1). Exceptions were respondents with severe dental anxiety and those reporting poor dental health for whom symptomatic visiting was the most common pattern. For many of the variables, there was a tendency that the same groups/categories had relatively high proportions in both extremes of dental visiting patterns—reporting symptomatic visits only as well as visiting regularly more than once a year. This was the case for elderly respondents, respondents without higher education, those reporting oral pain, and poor dental health, as well as for those with severe periodontitis. Men had less frequent dental visits than women. Respondents with a difficult financial situation and those with three or more decayed teeth less frequently reported regular annual visits and a high proportion reported a symptomatic visiting pattern (Table 1). Having regular visits with longer intervals than 2 years was most common in the youngest age group.

**Table 1:** Dental visiting pattern.

	Dental visiting patterns						
	Regular >1/year % (*n*)	Regular 1/year % (*n*)	Regular 1/2nd year % (*n*)	Regular <1/2nd year % (*n*)	Symptomatic % (*n*)	Total *n*	*p* a
All participants	21 (4381)	53 (10,903)	9 (1916)	6 (1293)	10 (2099)	20,592	
Sociodemographic variables							
Sex							
Female	23 (2449)	54 (5895)	9 (1021)	5 (591)	8 (844)	10,800	<.001
Male	20 (1932)	51 (5008)	9 (895)	7 (702)	13 (1255)	9792	
Age group (years)							
40–54	14 (1286)	55 (5190)	13 (1189)	9 (805)	10 (918)	9388	<.001
55–69	27 (2147)	54 (4264)	7 (574)	5 (388)	7 (543)	7016	
≥70	29 (948)	44 (1449)	5 (153)	3 (100)	19 (683)	3288	
Education							
No higher education	23 (2333)	51 (5184)	7.5 (772)	6 (610)	13 (1334)	10,233	<.001
Higher education	20 (1967)	56 (5582)	13 (1128)	7 (660)	7 (676)	10,013	
Finances							
Good	22 (3109)	56 (8103)	9 (1303)	6 (818)	8 (1101)	14,434	<.001
Average	21 (1101)	47 (2540)	10 (545)	8 (409)	14 (770)	5365	
Difficult	22 (158)	31 (227)	9 (65)	9 (64)	29 (208)	721	
Dental anxiety							
MDAS ≤ 18	22 (4217)	54 (10,564)	9 (1835)	6 (1187)	9 (1752)	19,555	<.001
MDAS ≥ 19	12 (71)	28 (163)	6 (36)	13 (77)	40 (231)	578	
Self‐reported oral health variables							
Oral pain last 4 weeks							
No	21 (4054)	53 (10,255)	9 (1800)	6 (1211)	10 (1920)	19,240	<.001
Yes	26 (168)	45 (296)	10 (62)	8 (49)	12 (77)	652	
Dental health							
Poor	28 (550)	24 (464)	5 (106)	8 (164)	34 (669)	1953	<.001
Moderate	25 (1785)	47 (3363)	9 (642)	8 (646)	11 (755)	7091	
Good	18 (1984)	62 (6968)	10 (1151)	5 (563)	4 (500)	11,166	
Clinical oral health variables							
Attended dental exam	22.3 (859)	53.1 (2051)	8.4 (326)	6.4 (248)	9.7 (375)	3939	
Decayed teeth (D3–5)							
0	23 (692)	55 (1645)	8 (241)	5 (163)	9 (262)	3003	<.001
1	21 (103)	51 (248)	10 (49)	9 (45)	9 (42)	487	
2	15 (24)	48 (78)	10 (16)	12 (20)	15 (25)	163	
≥3	17 (21)	30 (37)	10 (13)	14 (17)	30 (37)	125	
Periodontitis							
No	11 (106)	62 (606)	11 (112)	8 (74)	8 (78)	976	<.001
Mild	17 (140)	60 (509)	11 (93)	7 (57)	6 (49)	848	
Moderate	29 (406)	51 (720)	6 (85)	6 (81)	8 (115)	1407	
Severe	45 (175)	32 (125)	6 (22)	6 (25)	11 (44)	391	

Abbreviation: MDAS, Modified Dental Anxiety Scale.

^a^ Differences between groups assessed with Pearson's *χ* ^2^ test.

### 3.2 Number of dental visits in the past 12 months

As having regular dental visits more than once a year seemed to be associated with poorer oral health than less frequent regular visits, we did further analyses on the association between the number of dental visits in the preceding 12 months and sociodemographic and oral health variables (Table 2). Almost 17% reported three or more dental visits during the past year and the number of dental visits corresponded fairly well with the reported visiting pattern. Three or more dental visits were most common among respondents with a difficult financial situation, those reporting oral pain, poor oral health, and respondents with moderate or severe periodontitis (Table 2). More than 60% of those reporting three or more dental visits the previous year had moderate or severe periodontitis. Of the respondents with three or more carious teeth, 57% had no dental visits the preceding year (Table 2).

**Table 2:** Dental visits last 12 months.

	Number of dental visits last 12 months						
	0% (*n*)	1% (*n*)	2% (*n*)	3%–4% (*n*)	≥5% (*n*)	Total *n*	*p* a
	25 (4887)	38 (7490)	20 (3895)	12 (2268)	5 (997)	19,537	
Dental visiting pattern							
> 1/year	4 (161)	15 (581)	39 (1580)	29 (1149)	13 (532)	4003	<.001
1/year	12 (1172)	58 (5853)	19 (1953)	8 (852)	3 (303)	10,133	
1/2nd year	51 (937)	33 (601)	8 (140)	5 (99)	2 (43)	1820	
< 1/2nd year	74 (924)	14 (181)	5 (60)	4 (53)	3 (35)	1253	
Symptomatic	79 (1527)	9 (165)	5 (95)	4 (84)	3 (62)	1933	
Sociodemographic variables							
Sex							
Female	22 (2255)	39 (4015)	20 (2071)	12 (1269)	5 (558)	10,168	<.001
Male	28 (2632)	37 (3475)	20 (1824)	11 (999)	5 (439)	9369	
Age group (years)							
40–54	29 (2626)	42 (3831)	17 (1550)	9 (801)	4 (367)	9175	<.001
55–69	20 (1465)	37 (2749)	23 (1692)	15 (1097)	6 (460)	7463	
≥70	27 (796)	31 (910)	23 (653)	13 (370)	6 (170)	2899	
Education							
No higher education	27 (2573)	35 (3370)	20 (1940)	12 (1180)	5 (517)	9580	<.001
Higher education	23 (2206)	42 (4064)	20 (1914)	11 (1059)	5 (468)	9711	
Finances							
Good	23 (3066)	41 (5566)	21 (2791)	12 (1587)	4 (578)	13,588	<.001
Average	29 (1439)	34 (1697)	19 (956)	11 (567)	7 (330)	4989	
Difficult	40 (261)	20 (135)	15 (101)	14 (91)	11 (71)	659	
Dental anxiety							
MDAS ≤ 18	23 (3564)	40 (6371)	21 (3292)	12 (1920)	5 (811)	15,958	<.001
MDAS ≥ 19	31 (681)	35 (766)	17 (379)	11 (238)	5 (106)	2170	
Self‐reported oral health variables							
Oral pain last 4 weeks							
No	25 (4490)	39 (7025)	20 (3606)	11 (2067)	5 (903)	18,079	<.001
Yes	22 (138)	31 (118)	21 (134)	17 (106)	10 (50)	637	
Dental health							
Poor	41 (775)	16 (294)	13 (252)	15 (285)	14 (267)	1873	<.001
Moderate	25.3 (1713)	31.4 (2124)	22 (1456)	15 (1020)	7 (447)	6760	
Good	21 (2223)	47 (5027)	20 (2163)	9 (945)	3 (274)	10632	
Clinical oral health variables							
Attended dental exam	25 (887)	38 (1373)	21 (751)	10 (434)	5 (173)	3618	
Decayed teeth (D3–5)							
0	23 (644)	39 (1088)	22 (615)	12 (325)	5 (134)	2806	<.001
1	26 (122)	38 (175)	17 (77)	14 (64)	6 (27)	465	
2	32 (49)	33 (50)	19 (29)	13 (20)	3 (4)	152	
≥3	57 (56)	26 (31)	13 (16)	10 (12)	4 (5)	120	
Periodontitis							
No	26 (244)	45 (419)	18 (170)	7 (69)	2 (21)	923	<.001
Mild	25 (203)	41 (337)	20 (161)	10 (80)	4 (32)	813	
Moderate	19 (245)	37 (481)	24 (308)	14 (188)	6 (80)	1302	
Severe	27 (101)	19 (71)	24 (91)	21 (77)	9 (33)	373	

Abbreviation: MDAS, Modified Dental Anxiety Scale.

^a^ Differences between groups assessed with Pearson's *χ* ^2^ test.

The intended outcome of regular dental visits is to promote oral health. An alternative to regular screenings is to seek help for symptoms, typically pain, which could be rational if pain is a reliable predictor of disease. We performed two logistic regression analyses to estimate the associations of dental visiting patterns and oral pain with caries and periodontitis when controlling for sex, age, personal finances, and dental anxiety. The first model had dentine caries as the outcome (Table 3) and the second moderate or severe periodontitis (Table 4). Having a symptomatic visiting pattern predicted both caries and periodontitis. There was no significant difference in the prevalence of caries or periodontitis between those visiting the dentist annually or every other year, whereas more than 2 years between dental visits increased the likelihood of carious lesions. Oral pain was not significantly associated with either caries or periodontitis. The characteristics that had a significant association with dentine caries were high dental anxiety, symptomatic, or no visits to the dentist, being a man, and being in the youngest age group (Table 3). The characteristics that had a significant association with moderate or severe periodontitis were high age, visiting the dentist more than once a year, having a symptomatic dental visiting pattern and being a man (Table 4).

**Table 3:** Logistic regression models for dentine caries.

	Dentine caries (*n* = 3467)	
Variables	Unadjusted OR (95% CI)	Adjusted OR (95% CI)
Sex		
Female	Ref	Ref
Male	**1.54 (1.35, 1.77)**	**1.53 (1.32, 1.78)**
Age groups		
40–54	Ref	Ref
55–69	**0.73 (0.63. 0.85)**	**0.79 (0.67, 0.93)**
≥70	**0.61 (0.50, 0.75)**	**0.65 (0.52, 0.81)**
Finances		
Good	Ref	Ref
Average	1.14 (0.97, 1.34)	1.09 (0.92, 1.29)
Difficult	**1.72 (1.19, 2.48)**	1.48 (0.99, 2.20)
Dental anxiety		
MDAS ≤ 18	Ref	Ref
MDAS ≥ 19	**2.40 (1.55, 3.71)**	**2.18 (1.36, 3.49)**
Oral pain		
No	Ref	Ref
Yes	1.17 (0.77, 1.77)	1.11 (0.72, 1.71)
Dental visits		
1/year	Ref	Ref
>1/year	0.99 (0.83, 1.19)	1.01 (0.84, 1.22)
1/2nd year	1.62 (0.98, 1.62)	1.16 (0.89, 1.52)
<1/2nd year	**1.97 (1.50, 2.58)**	**1.77 (1.34, 2.35)**
Symptomatic	**1.36 (1.08, 1.73)**	**1.36 (1.05, 1.77)**
Constant		0.36
Hosmer and Lemeshow tests		*p* > .05
*χ* ^2^ (14, *N* = 3939)		101.16, *p* < .01
Nagelkerke *R* ^2^		0.04

*Note*: Numbers in bold indicate statistically significant differences to the reference group (p < 0.01).

Abbreviations: CI, confidence interval; MDAS, Modified Dental Anxiety Scale; OR, odds ratio.

**Table 4:** Logistic regression models for moderate and severe periodontitis.

	Moderate or severe periodontitis (*n* = 3407)	
Variables	Unadjusted OR (95% CI)	OR (95% CI)
Sex		
Female	Ref	Ref
Male	**1.48 (1.30, 1.69)**	**1.59 (1.37,1.84)**
Age groups		
40‐54	Ref	Ref
55‐69	**3.11 (2.68, 3.60)**	**3.08 (2.63,3.62)**
≥70	**5.16 (4.20, 6.33)**	**5.00 (3.99,6.30)**
Finances		
Good	Ref	Ref
Average	**1.19 (1.03, 1.39)**	**1.28 (1.08, 1.52)**
Difficult	1.01 (0.70, 1.46)	1.38 (0.91, 2.10)
Dental anxiety		
MDAS ≤ 18	Ref	Ref
MDAS ≥ 19	1.02 (0.65, 1.59)	1.30 (0.79, 2.16)
Oral pain		
No	Ref	Ref
Yes	0.10 (0.48. 1‐07)	0.98 (0.63, 1.53)
Dental visits		
1/year	Ref	Ref
>1/year	**3.12 (2.62, 3.71)**	**2.81 (2.32, 3.40)**
1/2nd year	**0.69 (0.54, 0.89)**	0.80 (0.60, 1.05)
<1/2nd year	1.07 (0.81, 1.40)	1.20 (0.89, 1.60)
Symptomatic	**1.65 (1.29, 2.12)**	**1.49 (1.11, 1.98)**
Constant		0.28
Hosmer and Lemeshow tests		*p* > .05
*χ* ^2^ (14, *N* = 3939)		547.33, *p* < .01
Nagelkerke *R* ^2^		0.20

*Note*: Numbers in bold indicate statistically significant differences to the reference group (*p* < .01).

Abbreviations: CI, confidence interval; MDAS, Modified Dental Anxiety Scale; OR, odds ratio.

## 4 DISCUSSION

As oral health improves in the population, it is important to assess if regular dental check‐ups are still warranted and if the frequency of dental visits reflects the oral health. In the present study, we have explored the associations between dental visiting patterns, sociodemographic variables, oral pain, and subjective, and clinical oral health measures, with emphasis on the characteristics of the high utilizers of dental services.

Ninety percent of the respondents in the present study reported a regular dental visiting pattern with varying frequencies, the most common being annual visits. Many studies have found oral health benefits from a regular dental visiting pattern as opposed to a symptomatic visiting pattern where one seeks help upon a subjective need (Aldossary et al., 2015; Astrom et al., 2014; Crocombe et al., 2012; Thomson et al., 2010). Thus, regular dental visits are generally considered a preventive measure. The definition of regular varies between studies, but some use a combination of reporting regular dental visits and having attended a dentist during the past 12 months. A Cochrane review assessing the optimal recall interval for dental check‐ups found little or no difference in caries, gingival bleeding, and oral health‐related quality of life between risk‐based and 24‐month intervals of dental check‐ups for adults over a 4‐year period (Fee et al., 2020). In our regression analyses, we found that having a symptomatic dental visiting pattern was associated with having both dentin caries and periodontitis, supporting that regular visits are associated with oral health benefits. We found no statistically significant differences in the odds of having caries or periodontitis between those reporting 12‐ and 24‐month intervals, whereas having longer than 24‐month intervals between visits was associated with higher odds of having caries.

In bivariate analyses, we found many of the same characteristics associated with the most frequent and symptomatic dental visiting pattern, such as being elderly, without higher education, reporting oral pain, having poor dental health, and having severe periodontitis. Furthermore, having more than one regular dental visit per year was strongly associated with having moderate or severe periodontitis in multivariate regression analyses. Among the respondents included in the clinical examination, about 70% of those who reported regular dental visits more than once a year had moderate or severe periodontitis. Systematic periodontal treatment typically involves multiple visits, and as periodontitis is a chronic disease, systematic treatment is often followed by a maintenance phase to keep the disease at bay. Although there is no general agreement on optimal recall intervals during the periodontal maintenance phase, more frequent than yearly visits are often recommended in published literature (Farooqi et al., 2015). This aligns well with our findings that a large proportion of those with more than yearly regular dental visits had moderate or severe periodontitis. Thus, our findings suggest that individuals having regular dental visits with <12 months intervals define a group with particular oral problems, for whom the frequent visits seem to be based on risk assessment or treatment needs. This is important to account for in epidemiological studies, where participants with an annual dental visiting pattern, who typically show advantageous oral health measures, should be separated from those having a more frequent dental visiting practice.

Toothache is the most common reason for oral pain (Renton, 2011) and the reason for most acute consultations in dentistry (Anderson & Thomas, 2003; Farmakis et al., 2016). In the current study, the bivariate analyses demonstrated that reports of oral pain significantly differed across the use of dental health services. Respondents who experienced oral pain more frequently reported three or more dental visits within the past 12 months than those who did not experience oral pain. It is important to notice that pain was recorded for the past 4 weeks, whereas the number of dental visits was reported for the past 12 months. Thus, our results may underestimate the association between dental visits and oral pain, as pain before 4 weeks of the study may have caused acute dental visits with treatment that has resolved the pain. Oral pain was not a significant predictor of caries or periodontitis in the logistic regression analyses, perhaps demonstrating the predominantly silent progression of both diseases. It should be noted that the prevalence of oral pain among the participants allocated to the dental examination was low (3%). Either way, pain seems to be an unreliable indicator of caries and marginal periodontitis.

Although Norway is a wealthy country that provides publicly financed health services and welfare solutions for the population, dental health care is generally paid out‐of‐pocket for adults, rendering these services and oral health vulnerable to disparities (Northridge et al., 2020). Almost 30% of those reporting a difficult financial situation had a symptomatic dental visiting pattern and 40% had not visited a dentist during the past 12 months. In contrast, only 8% of those with good finances reported a symptomatic visiting pattern and almost 80% reported at least one dental visit during the past 12 months. Nevertheless, a higher proportion of respondents with a difficult financial situation reported three or more dental visits during the past year compared to those with good finances. This suggests that a difficult financial situation is a barrier to seeking regular dental care, which may increase the risk of accumulating substantial and advanced treatment needs. Once identified, these may in turn require multiple visits and be more costly than regular dental visits focusing on preventive measures. This interpretation somewhat conflicts with a previous study among Norwegian adults that found a very small difference in the demand for dental services according to household income (Grytten et al., 2012). In that study, the demand for dental services was defined as having visited a dentist during the past 12 months. However, the need for dental treatment is not the same as having visited a dentist during the last year; dental treatment might be an unaffordable luxury for many irregular attenders. Also, household income does not necessarily translate into the reported financial situation, which will be influenced by numerous other factors than income, such as mortgages, care for children, and passive and portfolio income. These factors often differ significantly between and within this study's age‐groups. In multivariate regressions, a difficult personal financial situation was not significantly associated with caries or periodontitis. However, when performing the analysis stepwise, poor finances were significantly associated with dentin caries before adding dental visits to the model (not reported), perhaps reflecting that the financial situation affects dental caries by influencing the visiting pattern.

### 4.1 Limitations

The study is cross‐sectional; thus, we cannot conclude causality in associations. Central physical, biological, behavioral, and lifestyle‐related predictors of caries and periodontitis (Du et al., 2018; Selwitz et al., 2007) were not included in this study, influencing the regression models’ ability to predict the variance of diseases. However, the regression analyses were performed to assess the odds ratio and 95% CI of dental visits and oral pain on disease in the presence of sociodemographic variables and dental anxiety. Considering both the intention and the overall goodness of fit of the models, they were considered adequate despite the relatively low Nagelkerke *R* ^2^ values.

Another limitation of this study relates to the power of some measures in the regression analyses; the low number of individuals with high dental anxiety or a difficult financial situation among those included in the dental examination influences the statistical power of these measures. Nevertheless, the proportion reporting high dental anxiety or a difficult financial situation was similar among those included in the dental examination and in the complete Tromsø 7 cohort, suggesting representativeness. We also want to emphasize that we lack information on what type of screening or treatment was provided in the reported dental visits. However, the question “do you visit the dentist regularly?” suggests visits for screening or prevention, whereas a high number of actual visits in the past 12 months is more likely to reflect a significant disease burden.

Many of our measures are self‐reported and may be prone to social desirability bias or extreme responding bias. The study may also be affected by selection bias. Although the entire population 40 years or older in Tromsø municipality were invited, it is likely that persons with severe somatic or psychiatric illnesses, drug abuse disorders or immobilized or institutionalized persons had a low response rate.

## 5 CONCLUSIONS

Regular dental visits once a year or every second year were associated with the best dental health in terms of the absence of caries and severe periodontal disease. Dental visits more than once a year were strongly associated with periodontitis, and the most frequent dental visitors and the symptom‐driven visitors shared many characteristics. Oral pain was not a reliable predictor of caries or periodontal disease. As the dental visiting intervals were well aligned with oral health measures, our findings do not suggest that the dentist's economic interest is an important determinant of recall intervals in this population. Furthermore, our findings support the recommendation of regular dental visits to promote oral health.

## AUTHOR CONTRIBUTIONS

Hege Nermo and Elin Hadler‐Olsen contributed equally to study conception and design, data analyses and interpretation, and writing of the manuscript.

## CONFLICT OF INTEREST STATEMENT

The authors declare no conflict of interest.

## ETHICS STATEMENT

The Tromsø study was approved by the Norwegian Data Protection Authority (14/01463‐8/CGN) and the Regional Committee for Medical and Health Research Ethics approved the present study (320778). All study participants provided written, informed consent to participate in the Tromsø study.

## Data Availability

Data may be obtained from a third party and are not publicly available. The legal restriction on data availability is set by the Tromsø Study Data and Publication Committee to control data sharing, including publication of data sets with the potential of reverse identification of de‐identified sensitive participant information. The data can be made available from the Tromsø study upon application to the Tromsø Study Data and Publication Committee. E‐mail: tromsous@uit.no.

## References

[cre2753-bib-0002] Aldossary, A. , Harrison, V. E. , & Bernabé, E. (2015). Long‐term patterns of dental attendance and caries experience among British adults: A retrospective analysis. European Journal of Oral Sciences, 123(1), 39–45. 25521216 10.1111/eos.12161

[cre2753-bib-0003] Amarante, E. , Raadal, M. , & Espelid, I. (1998). Impact of diagnostic criteria on the prevalence of dental caries in Norwegian children aged 5, 12 and 18 years. Community Dentistry and Oral Epidemiology, 26(2), 87–94. 9645401 10.1111/j.1600-0528.1998.tb01933.x

[cre2753-bib-0004] Anderson, R. , & Thomas, D. W. (2003). ‘Toothache stories’: A qualitative investigation of why and how people seek emergency dental care. Community Dental Health, 20(2), 106–111. 12828271

[cre2753-bib-0005] Åstrøm, A. N. , Ekback, G. , Ordell, S. , & Nasir, E. (2014). Long‐term routine dental attendance: Influence on tooth loss and oral health‐related quality of life in Swedish older adults. Community Dentistry and Oral Epidemiology, 42(5), 460–469. 24712734 10.1111/cdoe.12105

[cre2753-bib-0006] Crocombe, L. A. , Broadbent, J. M. , Thomson, W. M. , Brennan, D. S. , & Poulton, R. (2012). Impact of dental visiting trajectory patterns on clinical oral health and oral health‐related quality of life. Journal of Public Health Dentistry, 72(1), 36–44. 22316176 10.1111/j.1752-7325.2011.00281.x

[cre2753-bib-0007] Du, M. , Bo, T. , Kapellas, K. , & Peres, M. A. (2018). Prediction models for the incidence and progression of periodontitis: A systematic review. Journal of Clinical Periodontology, 45(12), 1408–1420. 30394558 10.1111/jcpe.13037

[cre2753-bib-0008] Eke, P. I. , Page, R. C. , Wei, L. , Thornton‐Evans, G. , & Genco, R. J. (2012). Update of the case definitions for population‐based surveillance of periodontitis. Journal of Periodontology, 83(12), 1449–1454. 22420873 10.1902/jop.2012.110664 PMC6005373

[cre2753-bib-0009] Farmakis, E.‐T. R. , Palamidakis, F. D. , Skondra, F. G. , Nikoloudaki, G. , & Pantazis, N. (2016). Emergency care provided in a Greek dental school and analysis of the patients’ demographic characteristics: A prospective study. International Dental Journal, 66(5), 280–286. 27302884 10.1111/idj.12245 PMC9376644

[cre2753-bib-0010] Farooqi, O. A. , Wehler, C. J. , Gibson, G. , Jurasic, M. M. , & Jones, J. A. (2015). Appropriate recall interval for periodontal maintenance: A systematic review. Journal of Evidence Based Dental Practice, 15(4), 171–181. 26698003 10.1016/j.jebdp.2015.10.001 PMC4848042

[cre2753-bib-0011] Fee, P. A. , Riley, P. , Worthington, H. V. , Clarkson, J. E. , Boyers, D. , & Beirne, P. V. (2020). Recall intervals for oral health in primary care patients. The Cochrane Database of Systematic Reviews, 10, 004346. 10.1002/14651858.CD004346.pub5 PMC8256238 33053198

[cre2753-bib-0012] Frencken, J. E. , Sharma, P. , Stenhouse, L. , Green, D. , Laverty, D. , & Dietrich, T. (2017). Global epidemiology of dental caries and severe periodontitis–a comprehensive review. Journal of Clinical Periodontology, 44, S94–S105. 28266116 10.1111/jcpe.12677

[cre2753-bib-0013] Grytten, J. , Holst, D. , & Skau, I. (2012). Demand for and utilization of dental services according to household income in the adult population in Norway. Community Dentistry and Oral Epidemiology, 40(4), 297–305. 22239170 10.1111/j.1600-0528.2011.00659.x

[cre2753-bib-0014] Grytten, J. , Listl, S. , & Skau, I. (2022). Do Norwegian private dental practitioners with too few patients compensate for their loss of income by providing more services or by raising their fees? Community Dentistry and Oral Epidemiology, 1–8. 10.1111/cdoe.12750 35616472

[cre2753-bib-0015] Grytten, J. , & Skau, I. (2021). Improvements in Dental Health and Dentists' Workload in Norway, 1992 to 2015. International Dental Journal, 72(3), 399–406. 34479721 10.1016/j.identj.2021.07.004 PMC9275092

[cre2753-bib-0016] Hadler‐Olsen, E. , & Jönsson, B. (2021). Oral health and use of dental services in different stages of adulthood in Norway: A cross sectional study. BMC Oral health, 21(1), 257. 33985488 10.1186/s12903-021-01626-9 PMC8117586

[cre2753-bib-0017] Haugejorden, O. , & Klock, K. S. (2000). Avoidance of dental visits: The predictive validity of three dental anxiety scales. Acta Odontologica Scandinavica, 58(6), 255–259. 11196400 10.1080/00016350050217091

[cre2753-bib-0018] Holde, G. E. , Baker, S. R. , & Jönsson, B. (2018). Periodontitis and quality of life: What is the role of socioeconomic status, sense of coherence, dental service use and oral health practices? An exploratory theory‐guided analysis on a Norwegian population. Journal of Clinical Periodontology, 45(7), 768–79. 29681132 10.1111/jcpe.12906

[cre2753-bib-0019] Humphris, G. M. , Dyer, T. A. , & Robinson, P. G. (2009). The modified dental anxiety scale: UK general public population norms in 2008 with further psychometrics and effects of age. BMC Oral health, 9, 20. 19709436 10.1186/1472-6831-9-20 PMC2743651

[cre2753-bib-0020] Jönsson, B. , Holde, G. E. , & Baker, S. R. (2020). The role of psychosocial factors and treatment need in dental service use and oral health among adults in Norway. Community Dentistry and Oral Epidemiology, 48(3), 215–24. 32048318 10.1111/cdoe.12518

[cre2753-bib-0021] Kassebaum, N. J. , Smith, A. G. C. , Bernabé, E. , Fleming, T. D. , Reynolds, A. E. , Vos, T. , Murray, C. J. L. , Marcenes, W. , Abyu, G. Y. , Alsharif, U. , Asayesh, H. , Benzian, H. , Dandona, L. , Dandona, R. , Kasaeian, A. , Khader, Y. S. , Khang, Y. H. , Kokubo, Y. , Kotsakis, G. A. , … Yonemoto, N. (2017). Global, regional, and national prevalence, incidence, and Disability‐Adjusted life years for oral conditions for 195 countries, 1990–2015: A systematic analysis for the global burden of diseases, injuries, and risk factors. Journal of Dental Research, 96(4), 380–387. 28792274 10.1177/0022034517693566 PMC5912207

[cre2753-bib-0022] Mulic, A. , Tveit, A. B. , Stenhagen, K. R. , Oscarson, N. , Staxrud, F. , & Jönsson, B. (2020). The frequency of enamel and dentin caries lesions among elderly Norwegians. Acta Odontologica Scandinavica, 78(1), 6–12. 31267814 10.1080/00016357.2019.1634283

[cre2753-bib-0023] Norderyd, O. , Koch, G. , Papias, A. , Köhler, A. A. , Helkimo, A. N. , Brahm, C.‐O. , Lindmark, U. , Lindfors, N. , Mattsson, A. , Rolander, B. , Ullbro, C. , Gerdin, E. W. , & Frisk, F. (2015). Oral health of individuals aged 3‐80 years in Jönköping, Sweden during 40 years (1973‐2013): II. Review of clinical and radiographic findings. Swedish Dental Journal, 39(2), 69–86. 26529833

[cre2753-bib-0024] Northridge, M. E. , Kumar, A. , & Kaur, R. (2020). Disparities in access to oral health care. Annual Review of Public Health, 41, 513–35. 10.1146/annurev-publhealth-040119-094318 PMC7125002 31900100

[cre2753-bib-0025] Page, R. C. , & Eke, P. I. (2007). Case definitions for use in population‐based surveillance of periodontitis. Journal of Periodontology, 78(7 Suppl.), 1387–1399. 10.1902/jop.2007.060264 17608611

[cre2753-bib-0026] Petrenya, N. , Hopstock, L. A. , Holde, G. E. , Oscarson, N. , & Jonsson, B. (2022). Relationship between periodontitis and risk of cardiovascular disease: Insights from the Tromso Study. Journal of Periodontology, 93(9), 1353–1365. 35621303 10.1002/JPER.22-0004

[cre2753-bib-0027] Renton, T. (2011). Dental (Odontogenic) pain. Reviews in Pain, 5(1), 2–7. 10.1177/204946371100500102 PMC4590084 26527224

[cre2753-bib-0028] Selwitz, R. H. , Ismail, A. I. , & Pitts, N. B. (2007). Dental caries. The Lancet, 369(9555), 51–59. 10.1016/S0140-6736(07)60031-2 17208642

[cre2753-bib-0029] Steingrímsdóttir, Ó. A. , Engdahl, B. , Hansson, P. , Stubhaug, A. , & Nielsen, C. S. (2020). The graphical index of pain: A new web‐based method for high‐throughput screening of pain. Pain, 161(10), 2255–62. 32345913 10.1097/j.pain.0000000000001899 PMC7497597

[cre2753-bib-0030] Thomson, W. M. , Williams, S. M. , Broadbent, J. M. , Poulton, R. , & Locker, D. (2010). Long‐term dental visiting patterns and adult oral health. Journal of Dental Research, 89(3), 307–311. 20093674 10.1177/0022034509356779 PMC2821461

[cre2753-bib-0031] Øverland, S. K. A. , Vollset, S. E. , Kinge, J. M. , Skirbekk, V. , & Tollånes, M. C. (2018). Sykdomsbyrde i Norge 2016. Resultater fra Global Burden of Diseases, Injuries, and Risk Factors Study 2016 (GBD 2016). [Disease Burden in Norway 2016. Results from the Global Burden of Diseases, Injuries, and Risk Factors Study 2016 (GBD 2016)]. Folkehelseinstituttet.

